# The Importance of Fertility Preservation Counseling in Patients with Gynecologic Cancer

**Published:** 2017

**Authors:** Salvatore Giovanni Vitale, Valentina Lucia La Rosa, Agnese Maria Chiara Rapisarda, Antonio Simone Laganà

**Affiliations:** 1- Unit of Gynecology and Obstetrics, Department of Human Pathology in Adulthood and Childhood “G. Barresi”, University of Messina, Messina, Italy; 2- Unit of Psychodiagnostics and Clinical Psychology, University of Catania, Catania, Italy; 3- Department of General Surgery and Medical Surgical Specialties, University of Catania, Catania, Italy

It is estimated that gynecologic cancer has an incidence of 17% in the world (1). The most common gynecologic cancer is endometrial cancer with an incidence of 53% (2, 3). Although in most cases endometrial cancer manifests during menopause, in 25% of cases it can affect women in premenopausal age and in 2% of cases under the age of 40 (2). The treatment of this type of gynecological cancer is usually surgical and includes hysterectomy and bilateral salpingo oophorectomy (4–6). Adjuvant chemotherapy and/or radiotherapy is recommended in cases at high risk of recurrence and in the later stages of cancer (2, 6).

Ovarian cancer is the second most common gynecological malignancy and the leading cause of death for gynecologic cancer in Western countries (2). In most cases, it is diagnosed in advanced stages and mainly affects women aged between 55 and 65. The treatment of ovarian cancer usually involves a combination of surgery and chemotherapy (7–9).

Cervical cancer is the second cancer in women worldwide. However, in Western countries, thanks to the diffusion of prevention campaigns through systematic screening program for women aged between 25 and 65, the incidence of this cancer has been greatly reduced (10). Cervical cancer is often diagnosed in reproductive age and surgical treatment may be placed alongside radiation therapy (2, 11).

Thanks to progress made in the field of gynecologic oncology, the survival rate for women with gynecologic cancer is greatly increased over the years. Consequently, a primary objective in these cases is to gradually improve the quality of life of patients. Indeed, the experience of a gynecological cancer has a very strong impact on the psychological well-being of women; surgical treatment and chemotherapy can impair female identity and also sexual functioning (2, 3, 12–14).

Several studies confirm that women with gynecologic cancer experience low levels of quality of life, anxiety and depressive symptoms, suicidal thoughts, feelings of anger and shame, and low self-esteem (1, 3, 12–17).

Moreover, when cancer affects women in child-bearing age, treatments can jeopardize reproductive capacity. The possible infertility due to cancer in women can be more devastating than the cancer itself and the possibility to have a child after cancer can be an important incentive in the therapeutic process (18–20).

In the light of these general considerations, techniques for fertility preservation in women with gynecologic cancer can be very important for the improvement of quality of life of these patients (19–21). In Italy, according to the Guidelines for the preservation of fertility in cancer patients published in 2003, a conservative therapy for fertility preservation may be proposed in case of good prognosis and only in the presence of close follow-up and in cancer centers with experience and adequate follow-up protocols (22).

Several international studies have shown that an adequate counseling about the fertility preservation treatments is associated with an improved quality of life of women who survive from a gynecological cancer (19–22). However, in many cases, there is not adequate information about this type of treatment. The aforementioned Guidelines recommend that reproductive counseling should be offered immediately after the cancer diagnosis in order to come to an agreement with the patient about the best fertility preservation technique which varies depending on the cancer and reproductive prognosis (22).

Reproductive counseling requires a multidisciplinary approach since it is necessary not only to choose the most appropriate preservation technique according to the prognosis and the risk of infertility related to cancer treatments, but also to assess the real motivation of woman to face a pregnancy and to become mother (22, 23).

Therefore, the presence of the psychologist, along with the oncologist and the specialist in reproductive medicine, is important to convey correct information to patients with gynecological cancer who wish to preserve their procreative capacity (24, 25).

In conclusion, it is appropriate to conduct further research about this topic in order to minimize the impact of cancer treatments on quality of life and psychological well-being of women with gynecological cancer.
